# Work-related musculoskeletal symptoms among rural middle-aged women in Korea: a qualitative photovoice study

**DOI:** 10.7586/jkbns.25.028

**Published:** 2025-08-19

**Authors:** Dajung Kim, Joo Hee Lee

**Affiliations:** 1 Department of Nursing, Cheju Halla University, Jeju, Korea; 2 College of Nursing, Woosuk University, Wanju, Korea

**Keywords:** Musculoskeletal pain, Women, Middle aged, Rural population, Qualitative research

## Abstract

**Purpose:**

This study explored the work-related musculoskeletal health management experiences of middle-aged female farmers in rural areas of South Korea, focusing on how they recognize, experience, and respond to chronic musculoskeletal symptoms in their daily agricultural work environment.

**Methods:**

A qualitative design using the photovoice method was employed with five participants aged 40~60 years, selected via purposive sampling. Participants were asked to take photographs reflecting their musculoskeletal symptoms and related health practices. Data collected through photographs taken by the participants, and group interviews were analyzed using Braun and Clarke's six-step thematic analysis.

**Results:**

The analysis revealed four central themes: (1) physical wear from daily work; (2) unseen and unacknowledged pain; (3) barriers to seeking musculoskeletal relief; and (4) adaptive self-management strategies.

**Conclusion:**

The study underscores the multidimensional challenges faced by middle-aged female farmers in managing musculoskeletal health. The photovoice method effectively captured their lived realities and provided context-specific implications for developing culturally sensitive, gender-informed, and community-based nursing interventions. These findings carry implications for rural health policy, nursing practice, and participatory research approaches in underserved populations.

## INTRODUCTION

Middle adulthood is widely recognized as a period of transition marked by physical aging and shifting social roles [1]. Middle-aged women are affected by metabolic and physiological changes related to postmenopausal hormonal changes and lifestyle, resulting in problems in the cardiovascular, osteoporosis, and endocrine systems. In particular, the prevalence of osteoporosis is reported to be approximately twice as high [2]. The decrease in bone density caused by hormonal changes due to menopause exacerbates muscle fatigue, and when combined with the “culture of putting up with it” in traditional agricultural societies, leads to the concealment of symptoms [3]. Previous quantitative studies have focused on measuring pain intensity and prevalence [4] but have failed to capture the impact of symptoms on individuals' daily lives or cultural contexts. This stems from the structural limitations of the quantitative paradigm, which reduces pain to “measurable objective data.”

Work-related musculoskeletal disorders (MSDs) are MSDs that occur in connection with occupational activities, with repetitive motions, improper work postures, heavy lifting, and prolonged static postures being the primary causes [5]. Agricultural workers are particularly vulnerable to MSDs, as they spend a significant portion of their working hours in physically demanding postures that place biomechanical stress on the neck, lower back, and lower extremities. According to Kee [6], more than half of farmers’ daily work involves activities that strain the cervical spine, lumbar region, and legs, contributing to a high prevalence of MSDs such as rotator cuff injuries, disc herniation, and knee joint degeneration.

Women in agriculture are more likely to experience musculoskeletal pain or disorders than men [7,8]. Women living in rural areas are burdened by agricultural production work, housework, raising children, and caring for parents, due to their dual roles as farmers and housewives [9]. As they juggle farm work, household duties, and caregiving, their pain affects not only the body but also daily life, family roles, and self-perception [7]. Musculoskeletal symptoms in middle-aged rural women are not solely caused by physical strain, but also influenced by social, psychological, and economic factors. Despite this complexity, previous research has largely relied on quantitative methods such as surveys and epidemiological data, which tend to isolate symptoms from the broader life context [3,4]. These approaches provide useful information on prevalence and risk factors but fail to capture the subjective, symbolic, and sociocultural dimensions of illness.

To explore the multidimensional nature of lived experiences, qualitative research methods are particularly well suited. However, the effectiveness of in-depth interviews can be influenced by participants’ comprehension, language proficiency, and expressive abilities. Participants with limited literacy or verbal fluency may find it difficult to respond to abstract or emotionally complex questions [10]. Photovoice is a participatory action research methodology that empowers participants to document their everyday realities through photography and engage in collective reflection, thereby overcoming the limitations of traditional qualitative interviews. By enabling individuals to select and express visually meaningful moments, photovoice contributes to the expression of implicit or unexpressed experiences that may be constrained by verbal communication or researcher-driven frameworks [11,12]. The application of photovoice to the exploration of musculoskeletal symptoms is a choice rooted in the essential characteristics of the research topic. This method comprehensively illuminates the interaction between body, environment, and culture among middle-aged women in rural areas, going beyond simple data collection to provide empowerment for participants and seeds for social change. Photovoice intuitively reveals the context of physical pain by visualizing daily struggles that are often difficult to verbalize. Through group discussions, participants interpret each other's photos collectively, uncovering shared meanings and cultural codes embedded in their daily lives. This process transforms personal experiences into insights that can be translated into collective awareness, community-level needs, and potential pathways for social action [13,14].

This study aims to explore how middle-aged female farmers perceive and manage their musculoskeletal health by utilizing the photovoice method to analyze the impact of musculoskeletal symptoms experienced in daily life and the coping strategies employed by participants. The research question posed is “What is the meaning of experience for musculoskeletal symptoms in middle-aged women working in agriculture?”.

## METHODS

### 1. Study design

This study is a qualitative study that uses the photovoice research method [13] to explore the underlying meanings of middle-aged women's experiences of musculoskeletal symptoms in agriculture. This study adheres to the Consolidated Standards of Reporting Qualitative Research guidelines.

### 2. Participants

Participants were female farmers aged 40 to 60 years, registered as members of an agricultural cooperative in Jeju-si, whose primary occupation and source of income was farming, and who had engaged in farming for at least 10 years. Recruitment was conducted by randomly selecting one agricultural cooperative, posting a recruitment notice, and directly promoting the study to interested individuals during the researcher’s on-site stay. Five participants were finally enrolled after they voluntarily agreed to participate, fully understanding the study’s purpose, procedures, confidentiality protections, and the process of interview recording and photograph submission (excluding personal information) (Table 1). The small sample size of five participants aligns with the recommended number of participants for photo-based research to facilitate in-depth participation, ethical participation, and group discussions [13].

### 3. Group discussion process

Group discussions were designed as collaborative time for participants to share, interpret, and reflect on their actual experiences through photographs and engage in reflection. The discussions were conducted based on the photovoice principles established by Wang and Burris [13] and the critical dialogue framework proposed by Wilson et al. [14].

The discussions were conducted using a semi structured set of open-ended questions, encouraging participants to freely express their interpretations and insights. The main questions were as follows:

• *Why did you take this photo?*

• *How do you think this photo reflects your experience or part of your life?*

• *What message did you want to convey through this photo?*

• *How do you think this photo expresses your feelings or thoughts?*

• *What do you think this photo says about the theme (e.g., health or care)?*

The final session served as a feedback and reflection meeting, where participants shared their overall impressions of the photovoice process and insights gained from the discussions.

### 4. Data collection

Data collection took place from September to October 2024. This period was selected to minimize seasonal variations that could influence participants' awareness of musculoskeletal symptoms, particularly avoiding the peak seasons of summer and winter. The photo voice process consisted of four structured sessions: one orientation session, two group interviews, and one final reflection meeting. The sessions were conducted in quiet meeting rooms at local agricultural cooperatives or community cafes with the participants' consent.

In the first session (orientation), the researcher explained the purpose of the study, ethical procedures, and the concept of photo voice. Participants were instructed on how to take meaningful photos reflecting their musculoskeletal pain and self-care practices. Guidelines included avoiding identifiable individuals or copyright-protected content, and sample photos unrelated to the research topic were used for training to prevent bias. Participants were encouraged to take meaningful photos from their daily lives without needing artistic skills. Participants submitted written consent forms at the end of the session. Following the orientation, participants were asked to take 5~6 photos with their smartphones according to two thematic guidelines (Session 2: “Experiences of musculoskeletal pain in daily life,” and Session 3: “Methods of managing or responding to musculoskeletal discomfort in daily life”). The number of photos was selected based on previous photovoice studies, which recommended capturing a manageable yet diverse set of photos to promote reflection and discussion [14,15]. Participants submitted their selected photos to the researcher via mobile messenger prior to the group interview. All interviews were audio-recorded with consent and transcribed verbatim for thematic analysis (Table 2).

### 5. Data analysis

The collected data were analyzed using Braun and Clarke's six-step thematic analysis framework [16]. This framework is suitable for identifying patterns and meanings across qualitative data. This method was chosen for its flexibility and rigor in analyzing empirical data rooted in participants' actual experiences. Researchers listened to the interview recordings at least three times and transcribed them verbatim. During this process, nonverbal cues such as sighs or hesitations were recorded in footnotes. The transcription data was read five times independently by two researchers (Step 1: Familiarization). Meaningful units related to participants' musculoskeletal symptoms, daily experiences, coping strategies, and access to medical services were systematically identified, and initial codes were generated using Excel (Step 2: Initial code generation). The 87 initial codes were reviewed and grouped to capture recurring concepts and patterns, resulting in four main themes and 12 subthemes (Step 3: Theme exploration). Themes were jointly refined and reviewed to ensure consistency within themes, differences between themes, and maintain authenticity through comparison with the original data (Step 4: Theme review). Two experts in rehabilitation medicine and women's health research validated the content of the main themes and subthemes. Each theme was defined and named using representative quotes from participants to explain its fundamental meaning (Step 5: Theme definition and naming). Finally, we presented 2~4 representative quotes for each theme to clarify the basis for participant selection (Step 6: Report writing). This six-step process was iterative and cyclical, with five feedback loops occurring between Steps 3 and 5.

### 6. Trustworthiness

The rigor of this qualitative study was established by applying Lincoln and Guba’s criteria [17], which include credibility, transferability, dependability, and confirmability. Credibility was ensured through purposive sampling of participants who could provide rich, detailed insights into musculoskeletal health management. Multiple group discussions utilizing the photovoice method facilitated in-depth data collection, while member checking during the final session allowed participants to verify and refine key findings. Transferability was supported by offering comprehensive descriptions of participant demographics, agricultural work contexts, and the surrounding socio-cultural environment, enabling readers to judge the relevance of the findings to other settings. Dependability was reinforced by systematically documenting the research process, including recruitment procedures, data collection and analysis methods, and researcher interactions. An audit trail was maintained to promote transparency and consistency.

Confirmability was strengthened by involving two independent researchers in data analysis to minimize individual bias and by engaging in continuous reflexivity through regular discussions and reflective journaling. The inclusion of direct participant quotations further enhanced the transparency and authenticity of the findings. Collectively, these strategies contributed to the methodological rigor of the study and ensured the trustworthiness of its results.

### 7. Ethical considerations

This study was approved by the Institutional Review Board of Korean Armed Forces Nursing Academy (KAFNA-2024-05-001). The participants were informed of the purpose, method, duration of the study, the recording of interviews, the management of the collected data, and the confidentiality of personal information, and their written consent was obtained. In addition, the participants were informed that they could withdraw from the study at any time.

## RESULTS

A total of 52 photographs were submitted by participants and discussed during two rounds of group interviews. Through thematic analysis, 87 initial codes were identified, which were refined into 13 subthemes and ultimately consolidated into four overarching themes. These themes captured participants' multifaceted experiences and coping strategies in managing musculoskeletal health: (1) physical wear from daily work, (2) unseen and unacknowledged pain, (3) barriers to seeking musculoskeletal relief, and (4) adaptive self-management strategies.

### 1. Physical wear from daily work- “*it feels like my back is collapsing*”

This theme highlights participants’ gradual physical deterioration from long-term, repetitive farm work. They didn't always see these feelings as medical problems, but they consistently described the discomfort and physical strain that built up from everyday work. Their stories reflected the physical nature of rural labor and how it affected their musculoskeletal system over time.

#### 1) Accumulated strain from repetitive work

Participants frequently described pain and tension as the result of repeated movements such as bending, lifting, and harvesting. These repetitive tasks led to sensations of fatigue that were integrated into their daily routines and often endured without formal treatment.

“*If I don't work today, there will be more work tomorrow, so I can't afford to be late. When the harvest is good, I feel a sense of fulfillment, but to have a good harvest, I have to work hard all year round, and doing the same thing every year can be exhausting. And above all, standing and using my body all day is physically demanding.”* (Participant 4)

**Figure 1.**
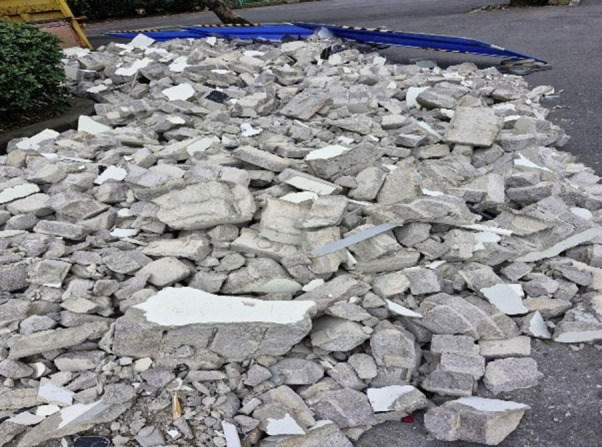
A figurative expression of chronic physical fatigue: “It feels like my back is collapsing.” (e.g., Photo by participant, used with consent)

“*Harvesting crops involves repeatedly bending and straightening my back countless times, which makes my back feel like it's about to break. It feels like bricks are collapsing, and it's both exhausting and painful*.” (Participant 5) (Figure 1)

#### 2) Improper work posture

Participants also explained that the nature of their work required them to adopt uncomfortable postures, which exacerbated musculoskeletal discomfort. In particular, prolonged bending, reaching above the head, and squatting were mentioned as major stress factors.

“*Especially in winter, my shoulders and arms feel stiff. I think it's because I have to keep lifting my arms while harvesting citrus fruits. It hurts even more when picking citrus fruits from trees or moving heavy boxes filled with harvested fruits... Especially in my case, when removing weeds, I have to bend and straighten my back repeatedly, which is really tough*.” (Participant 3)

“*Continuously picking tangerines from the trees causes my shoulders to ache. Additionally, when digging crops in the field, I must constantly move my arms without rest*.” (Participant 4) (Supplementary Figure 1)

#### 3) Physical limitations in daily life

The physical wear experienced during farm labor extended into participants’ personal lives, affecting their daily functioning and mobility. Participants described how their discomfort restricted activities they once enjoyed, instilling feelings of frustration, loss, and even shame.

“*I worked hard, but since I couldn't move my legs freely, I felt like I was useless... When I catch a cold, at least I can get better, but when my knees hurt, I can't go anywhere, which is frustrating and upsetting*.” (Participant 1)

**Figure 2.**
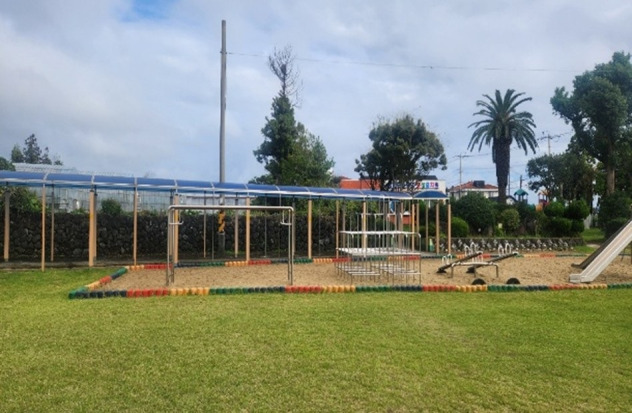
A distant memory of movement: “Just looking at the playground makes my knees hurt.” (e.g., Photo by participant, used with consent)

“*I usually feel pain in my knees and legs when I walk or run for long periods. But my grandchild is an elementary school student and runs around the playground kicking a ball and playing energetically. I wonder if I used to be like that too… So just seeing an open, spacious playground like that makes me think, ‘How much would my knees hurt if I ran there?’ It's scary just to look at it*.” (Participant 1) (Figure 2)

These reflections illustrate how physical wear disrupts daily living beyond the workplace, transforming ordinary activities into sources of fear, limitation, and emotional withdrawal. Pain was not isolated to the field but was experienced as a persistent companion that shaped their sense of identity and aging.

### 2. Unseen and unacknowledged pain- “*it’s a pain only I feel*”

This theme illustrates the emotional and relational dimension of musculoskeletal discomfort as experienced by participants. While the physical burden of labor was tangible to them, the lack of visible symptoms often led to emotional isolation and social misunderstanding. The women struggled not only with their bodily limitations but also with the frustration of not being believed or supported in their pain—especially within the family or community context.

#### 1) Emotional burden of invisible pain

Participants often expressed that their pain was not taken seriously by others because it was not outwardly visible. This lack of recognition resulted in feelings of being misunderstood or even dismissed.

“*Musculoskeletal pain isn't visible on the surface, so no one understands it, but it's a pain only I feel*” (Participant 4).

This invisibility created a psychological distance between the participants and those around them. For instance, Participant 4 continued by describing her emotional reaction after hearing someone on the radio share a similar experience:

“*I heard this on the radio, and it sounded so much like my own story that I cried. And my shoulders are like wings, but I feel like my wings are broken. Once they're broken, they don't heal, and it's so painful*” (Participant 4).

The participant's metaphorical expression conveyed the depth of despair and the sense of irreversible damage not just physically, but emotionally.

#### 2) Lack of understanding within families and communities

Family members' indifference or skeptical attitudes further exacerbated emotional burdens. Some participants described how spouses or colleagues minimized their experiences, insisting that the pain was a natural part of aging and not a valid concern. One woman recalled a painful conversation with her husband:

“*I tell him I'm in pain, but my middle-aged husband says, ‘You could be in pain.’ Those words really hurt. We've been farming together all our lives, and now that my body is breaking down, it feels like he's taking someone else's side*” (Participant 5).

These feelings reveal the social dimension of suffering, in which a lack of empathy acts as an additional layer of pain. The participants' experiences were emotionally painful beyond the physical difficulties, because they were constantly doubted or ignored by others regarding their inner state.

#### 3) Ambiguity in identifying the cause of pain

Some participants expressed confusion or uncertainty about the nature of their discomfort. They attempted to interpret their symptoms using familiar concepts, such as ‘frozen shoulder’ or ‘occupational disease.’

“*At first, I thought it was frozen shoulder. But when the symptoms didn't improve, I started to think it might be an occupational disease*.” (Participant 3).

Another participant emphasized how the pain progresses throughout the day:

“*The pain is worse by the end of the day than in the morning. When I'm working, I'm too busy to notice the pain, but by the end of the day, I realize how severe it is. But maybe it's just getting worse as I get older*.” (Participant 5)

These examples demonstrate that pain is experienced through complex interactions between work, aging, and emotions. Because the causes of pain are unclear, women's ability to express their discomfort becomes even more complicated, reinforcing invisible pain and self-doubt.

### 3. Barriers to seeking musculoskeletal relief “*Even going to the hospital feels faraway*”

This theme addresses the structural and practical obstacles participants faced when attempting to seek care for musculoskeletal discomfort. Although they recognized the importance of managing their symptoms, participants often encountered barriers related to geographical distance, time constraints, and limited healthcare infrastructure. As a result, musculoskeletal symptoms were frequently left unaddressed or managed through personal coping strategies rather than professional care.

#### 1) Practical barriers in daily life

Living in rural areas meant that access to specialized healthcare facilities was limited. Participants described the long distances they had to travel to see a doctor, often relying on others for transportation. This was further complicated by high costs, busy farming schedules, and the need to prioritize work over health.

“*There are no large hospitals here, only small ones, so if I need proper care, I have to go far, and that means high costs and long travel times*..." (Participant 3)

“*Since this is a rural area, I can't walk to the hospital. I have to take a car, but I can't drive. If my husband can drive me, that's good, but when he can't, I have to take the bus to the city. Transportation isn't as convenient here as it is in the city, so it's difficult*.” (Participant 2)

These logistical and financial burdens led to delayed or avoided treatment, resulting in symptoms being tolerated rather than addressed.

#### 2) Unclear expectations around telemedicine

Some participants had heard of telemedicine but were unsure of its relevance to their situation, especially regarding musculoskeletal problems. While they welcomed the idea of greater accessibility, they expressed skepticism about whether virtual consultations could adequately address their physical issues.

“*I've heard about meeting a doctor online. I really envy that. I wish medical facilities were better developed for people like us who live far from hospitals. I wish there was a way for people like us who live far away from hospitals to see a doctor and get treatment over the phone or computer*.” (Participant 2)

“*Since it's hard to go to the hospital, I heard there's a way to see a doctor remotely. Can prescriptions also be done that way? Of course, physical therapy requires an in-person visit, but such a system is really needed in rural areas*.” (Participant 3)

These perspectives reflect a desire for access to healthcare rather than specific technical requirements. Participants said that telemedicine would be helpful, but emphasized that it should be tailored to the needs of rural residents, particularly those with long-term physical discomfort.

#### 3) Demand for community-based health support

Participants emphasized the value of community health education programs and preventive healthcare that could be provided by local health centers or public health offices. They responded positively to group-based services tailored to physical needs and home exercise guidelines.

“*I've participated in a few health programs at the public health center, and I've been doing what I learned there whenever I have time. Farming doesn’t allow for much free time, especially during the busy harvest season. So, I'd like to attend educational programs like this during my free time. The stretching exercises for knee pain were really helpful*.” (Participant 1)

“*I feel like I should start managing my health before I get older, but I don’t know where to start. I wish someone could coach me. There’s so much information on my phone, and it all seems similar, so I don’t know what to look for or which information to trust*.” (Participant 3)

The absence of timely and relevant care leads to a cycle of self-management, uncertainty, and ultimately giving up. Participants reflect a desire for integrated, accessible, and culturally sensitive support that includes their physical labor, schedules, and lifestyles.

#### 4) Intermittent yet meaningful use of formal care

Although access to formal healthcare services such as physical therapy was often inconsistent and constrained, participants valued these opportunities as meaningful moments of temporary relief. Rather than expecting a cure, they viewed such care as a form of periodic support that complemented their self-devised pain management routines. Their use of formal care reflected a pragmatic approach shaped by seasonal work patterns, financial limitations, and time constraints.

“Going to physical therapy is really great. Even in the middle of endless farm work, getting physical therapy every now and then really lifts my spirits.” (Participant 4)”

**Figure 3.**
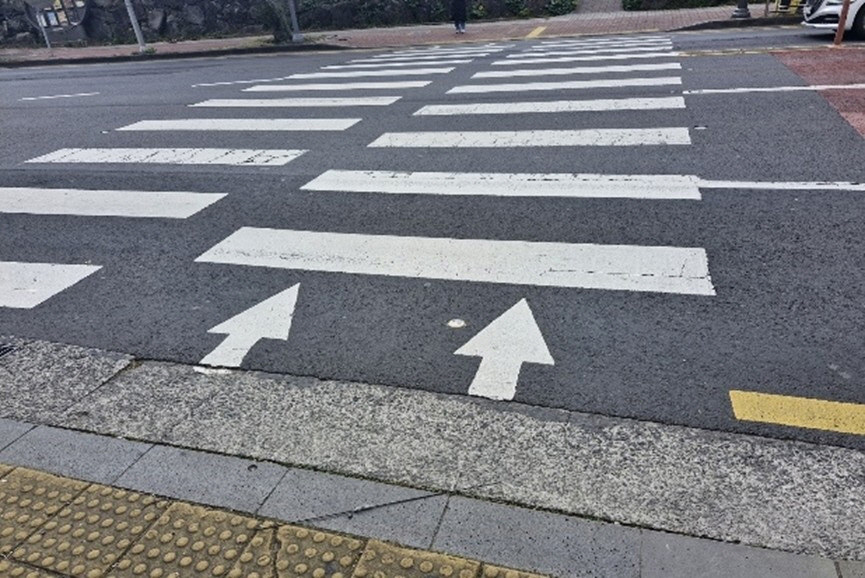
A pathway to relief and hope: “The path to physical therapy gives me a moment of comfort.” (e.g., Photo by participant, used with consent)

“I am very happy to see the crosswalk on my way to physical therapy at the hospital. Although I go to the hospital, I am so busy during the harvest season that I have no time to go, so I just endure the pain and work.” (Participant 2) (Figure 3)

### 4. Adaptive self-management strategies - “I endure it with what I have”

Faced with physical discomfort, limited medical access, and the demands of agricultural life, participants described developing personal, context-specific methods to manage their musculoskeletal symptoms. These strategies were not the result of formal medical advice but emerged organically from lived experiences, trial-and-error, and peer knowledge. They reflected the participants’ efforts to maintain their functional capacity while minimizing disruption to their daily responsibilities.

#### 1) Using warmth and physical relief techniques

“*I don't have much time, and it's a bit difficult for me to go to the hospital. So, I purchased this treatment device that I can use at home and use it frequently. When my bones or muscles hurt, I use massage. I sit in the massage chair and relax, and it seems to relieve my fatigue*.” (Participant 4)

“*I use warm compresses every day. Sometimes they’re much better than painkillers*.” (Participant 5) (Supplementary Figure 2)

These personal practices were accessible, low-cost, and emotionally reassuring—especially in a setting where formal care was not always an option.

#### 2) Dietary routines for musculoskeletal support

Some participants viewed nutrition as part of their pain management strategy and consciously chose foods they believed would strengthen their bones or improve their overall energy. These dietary habits were shaped by cultural knowledge and personal experiences and beliefs.

“*Since it’s related to bones, I eat more milk and anchovies. Eating is important, right?*” (Participant 4)

“*I try to eat well. If I don’t have energy, I can’t work. But now that I’m middle-aged, even if I eat and work, I don’t lose weight. Meals are important, but I'm worried because I can't eat everything I want*.” (Participant 5)

#### 3) Adequate rest and adjustment of work intensity

To manage symptoms and continue working, participants adjusted their posture, used assistive devices, and regulated their pace. These adaptations demonstrated a high level of body awareness and problem-solving skills to maintain a balance between health and productivity.

“*Before starting fieldwork, I rotate my waist and knees or stretch lightly. I try not to bend too much while working and use a small stool when needed. I bought a handcart with wheels and padded knee guards to reduce the strain on my body*.” (Participant 5)

Beyond task-specific adjustments, some participants also emphasized the importance of intentional rest and emotionally meaningful movement as a coping strategy. Rather than seeking complete avoidance of activity, they described how engaging in personally meaningful and self-directed movement helped offset discomfort and maintain emotional resilience.

“*Even before the trip, I sighed thinking about how much walking I'd have to do (laughs)... but it was fine. I felt better afterward, both in mood and pain. I've always loved traveling, so even though I’m in pain, I try to move and go places I enjoy. Even packing for a trip makes me happy*.” (Participant 1)

## DISCUSSION

This study utilized the photo-based participatory research method known as “photovoice” to explore how middle-aged female farmers in rural Korean regions perceive and manage musculoskeletal symptoms in their daily lives. As an art-based participatory action research method, photovoice has contributed to visualizing and expressing experiences that are often invisible or difficult to articulate verbally. By enabling participants to document their real-life experiences and discuss the photos together, this study revealed not only the physiological and occupational burdens associated with musculoskeletal issues but also their deeper psychological and sociocultural meanings. Four interrelated themes were identified: (1) physical wear from daily work, (2) unseen and unacknowledged pain, (3) barriers to seeking musculoskeletal relief, and (4) adaptive self-management strategies.

The first theme, “Physical fatigue caused by daily work,” emphasizes the cumulative physical strain experienced by middle-aged female farmers through long-term, repetitive agricultural labor. Participants described persistent pain in biologically vulnerable areas, particularly the lower back, shoulders, and knees, emphasizing that everyday farming tasks such as bending, lifting, and squatting exacerbate structural strain. These findings align with existing occupational health research [18], demonstrating that repetitive and unnatural postures are a primary cause of musculoskeletal tension among agricultural workers, especially in rural areas with limited ergonomic support. Participants did not conceptualize pain in biological terms but rather described it as a natural and inevitable byproduct of work. This reflects the process by which chronic physical discomfort becomes normalized and internalized in the absence of medical intervention or alternative labor strategies. A case illustrating this internalized perception is Figure 1, a photo submitted by a participant. The photo depicts a pile of collapsed cement blocks, and the participant described their pain as “the feeling of my back collapsing.” This image conveys the chronic deterioration and heaviness of the body through the metaphor of structural collapse. It symbolically represents not only the biomechanical consequences of repetitive stress but also the emotional fatigue accompanying prolonged physical wear and tear. The fragmented blocks symbolize a body that has lost its structural integrity, resonating with narratives of instability, vulnerability, and irreversible damage. This metaphor aligns with the biological understanding of musculoskeletal degeneration caused by accumulated mechanical stress, which can lead to microdamage, inflammation, and cartilage destruction over time [19,20]. Additionally, middle-aged women, especially those going through menopause, may experience exacerbated effects due to hormonal changes such as decreased estrogen levels [21-23]. Participants reported that this decline had affected their daily lives and self-concept. Tasks that were once routine became sources of anxiety or pain, and many participants described experiencing feelings of frustration, sadness, and worthlessness due to the decline in their physical abilities. These emotional responses demonstrate that physical decline is not merely a physical issue but a psychosocial problem deeply rooted in identity, work expectations, and the aging process. These findings suggest that interventions may be necessary that address both the physical and emotional dimensions of chronic occupational stress among rural women, grounded in a context that reflects biological understanding.

The second theme, “Unseen and unacknowledged pain,” addresses the emotional and relational aspects of musculoskeletal pain. Physical strain from agricultural work was a common reality, but participants frequently mentioned that their pain was not taken seriously. This was due to the absence of visible symptoms or cultural suppression of expressing pain. These findings align with previous qualitative research indicating that rural women internalize physical pain as a normal part of life in environments where hard labor and endurance are culturally idealized [24]. Bartley and Fillingim [25] reported that gender differences in pain sensitivity are partially mediated by hormonal influences and social factors. These findings support our results and emphasize the importance of recognizing chronic musculoskeletal pain as a multidimensional health issue rather than a simple physical disability. Such biases can also be found among healthcare providers. Paul-Savoie et al. [26] reported that nurses showed 38% less empathy and provided 42% less patient-centered care to patients with invisible pain conditions such as fibromyalgia compared to patients with visible disabilities. This highlights the existence of significant biases in clinical responses to pain without observable symptoms. Therefore, nursing interventions should include culturally sensitive communication strategies that acknowledge subjective experiences, particularly targeting populations at risk of emotional alienation and lack of social visibility. Recognizing and addressing the invisible burden of chronic pain is essential to improving quality of life in rural areas and ensuring more inclusive and person-centered care.

The third theme, “Barriers to seeking musculoskeletal relief,” highlights the structural and practical constraints that middle-aged female farmers face when seeking medical support management of musculoskeletal conditions. According to the Korea Rural Economic Institute [27], only 12.9% of medical institutions are located in rural areas, approximately 25% of rural counties have no emergency medical centers, and only 5.7% of doctors work in rural areas. This means that rural residents must continue to travel long distances to hospitals for specialized treatment, which is a major obstacle [28,29]. Institutional ethnographic studies have shown that these gendered expectations often prevent women from prioritizing their own health needs, and that they prioritize the well-being of family members and work responsibilities over self-care [30]. This dynamic was particularly evident in this study, where female agricultural workers endured considerable pain to meet labor demands during the farming season, often delaying or forgoing needed care. As shown in Figure 3, the crosswalk serves as a symbolic representation of the fragmented yet hopeful journey that participants undergo in seeking formal treatment for musculoskeletal symptoms. Participants revealed that clinic visits and rehabilitation therapy -albeit infrequent- contributed greatly to their psychological and physical relief. Participants’ interest in telehealth and community-based health services suggests the potential of alternative healthcare delivery models that can improve access to musculoskeletal care in underserved rural regions. In the context of musculoskeletal care, telemedicine cannot replace direct interventions such as physical therapy, but it can play an important complementary role through remote education, including posture correction, pain self-monitoring, home-based exercise guidelines, and medication counseling. Previous studies have shown that telerehabilitation can be an effective strategy for managing musculoskeletal symptoms in areas with limited access to healthcare services [31]. While this study provides valuable insight into the barriers to healthcare experienced by middle-aged rural women, further research is needed to develop sustainable strategies to address these regional disparities. Expanding gender-sensitive and biologically informed health policies can play an important role in improving health outcomes for diverse rural populations. The third theme provides valuable insights into the barriers to healthcare access faced by middle-aged rural women, but further research is needed to develop sustainable strategies to address these regional disparities. Expanding gender-sensitive and biologically appropriate health policies can play an important role in improving health outcomes for diverse rural populations.

The fourth theme, “Adaptive self-management strategies” identifies diverse and creative strategies developed by middle-aged women in rural areas with limited access to specialized medical services to overcome musculoskeletal discomfort. Voluntary implementation of personalized daily habits not only reflects high levels of body awareness and functional adaptation but also mirrors the economic and social realities of rural life, characterized by limited access to medical services and time constraints due to agricultural labor. This aligns with the coping strategies employed by individuals experiencing chronic low back pain [32]. Participants also described adjusting their work postures and using low-cost tools such as padded knee guards or small chairs to minimize physical strain. These ergonomic adaptations reflected embodied knowledge derived from peer sharing and experience rather than professional prescriptions. In another qualitative study, participants in a chronic pain management program at a primary care facility reported positive changes in pain management after participating in the program [33]. Therefore, it is likely that these strategies could make a positive difference in the lives of middle-aged female agricultural workers with chronic pain.

This study focuses on the unique health experiences of middle-aged female agricultural workers who face both biological vulnerabilities and occupational risks due to physically demanding labors. Despite the clear intersection of gender, occupation, and rural health disparities, there is limited research exploring the actual experiences of this group in rural settings with limited access to healthcare. This study contributes to the development of patient-centered and contextually appropriate nursing interventions for this marginalized population by providing detailed insights rooted in participants' everyday realities through a qualitative, photo-based, participant-centered approach. However, several limitations exist. First, the study results are based on a small, purposefully selected sample from a single rural area, limiting generalizability. Second, as with most qualitative research, participants' responses may be influenced by memory bias or social desirability bias. Third, the analysis focused on women did not consider other or overlapping issues that male or non-binary agricultural workers may face. Future studies should consider comparative or cross-sectional approaches that include variables such as gender identity, age, and cultural background. Finally, the absence of biological health indicators (e.g., bone density, inflammation markers) limits the analysis of the correlation between subjective pain experiences and objective physiological indicators. Mixed-method studies integrating qualitative data and clinical evaluations could deepen our understanding of musculoskeletal health.

## CONCLUSION

In conclusion, this study investigated the experiences of middle-aged female farmers in managing their musculoskeletal health using the photovoice method. The results revealed that participants endure chronic physical stress caused by repetitive and physically demanding labor and experience social isolation due to the lack of visibility of their pain. Additionally, they face significant structural barriers to accessing medical services. In response, they rely on daily adaptive strategies. These findings suggest the importance of community-based nursing interventions that integrate biological, psychosocial, and contextual perspectives on musculoskeletal discomfort. Advancing occupational health nursing requires a gender-responsive and contextually informed approach that acknowledges the complex intersections of gender, labor, and rural health disparities.

## Figures and Tables

**Table 1.** Demographic and Occupational Characteristics of Middle-aged Female Farmers (N=5)

Participant	Age (year)	Main tasks	Main symptom	Management strategy
P1	49	Fruit harvesting	Knee pain	Physical therapy, rest
P2	48	Mixed farming	Back pain	Physical therapy, patches
P3	58	Fruit harvesting	Back pain, shoulder pain	Physical therapy, rest
P4	57	Fruit harvesting	Shoulder pain, wrist pain	Massage, rest
P5	55	Mixed farming	Back pain, shoulder pain	Physical therapy, rest

**Table 2.** Structure and Content of Photovoice Sessions for Musculoskeletal Health Experiences

Session	Content	Time (min)
1	Orientation	90
	· Research objectives, introduction to photo voice, participant introductions, ethics training	
	· Providing specific guidelines and explanations on photo shooting criteria and methods, and educating participants on precautions to take during photo shooting	
	Completion of participant consent forms	
	· Guidance on selecting 5~6 photos to be used in the first and second group interviews	
	· Discussion of the schedule for the first group interview	
2	First group interview	90
	· Sharing the meaning of the selected photos with each participant	
	· Discussing the schedule for the second group interview	
3	Second group interview	90
	· Sharing the meaning of the selected photos with each participant	
	· Discussing the schedule for the final evaluation meeting	
4	Final evaluation meeting	60
	· Overall evaluation of program participation and sharing of impressions	

## Data Availability

Please contact the corresponding author for data availability.

## Supplementary Materials

Supplementary Figure 1. Photographs captured by participants to represent experiences of musculoskeletal pain in daily life.

Supplementary Figure 2. How participants manage musculoskeletal pain in daily life.
