# Supplementary material for: Work-related musculoskeletal symptoms among rural middle-aged women in Korea: a qualitative photovoice study
Source: J Korean Biol Nurs Sci. 2025 Aug 19;27(3):477–88. doi: 10.7586/jkbns.25.028 (PMC13268291; doi:10.7586/jkbns.25.028)
Supplement: Supplementary file 1 [file jkbns-25-028-Supplmentary-Figure-1.pdf]

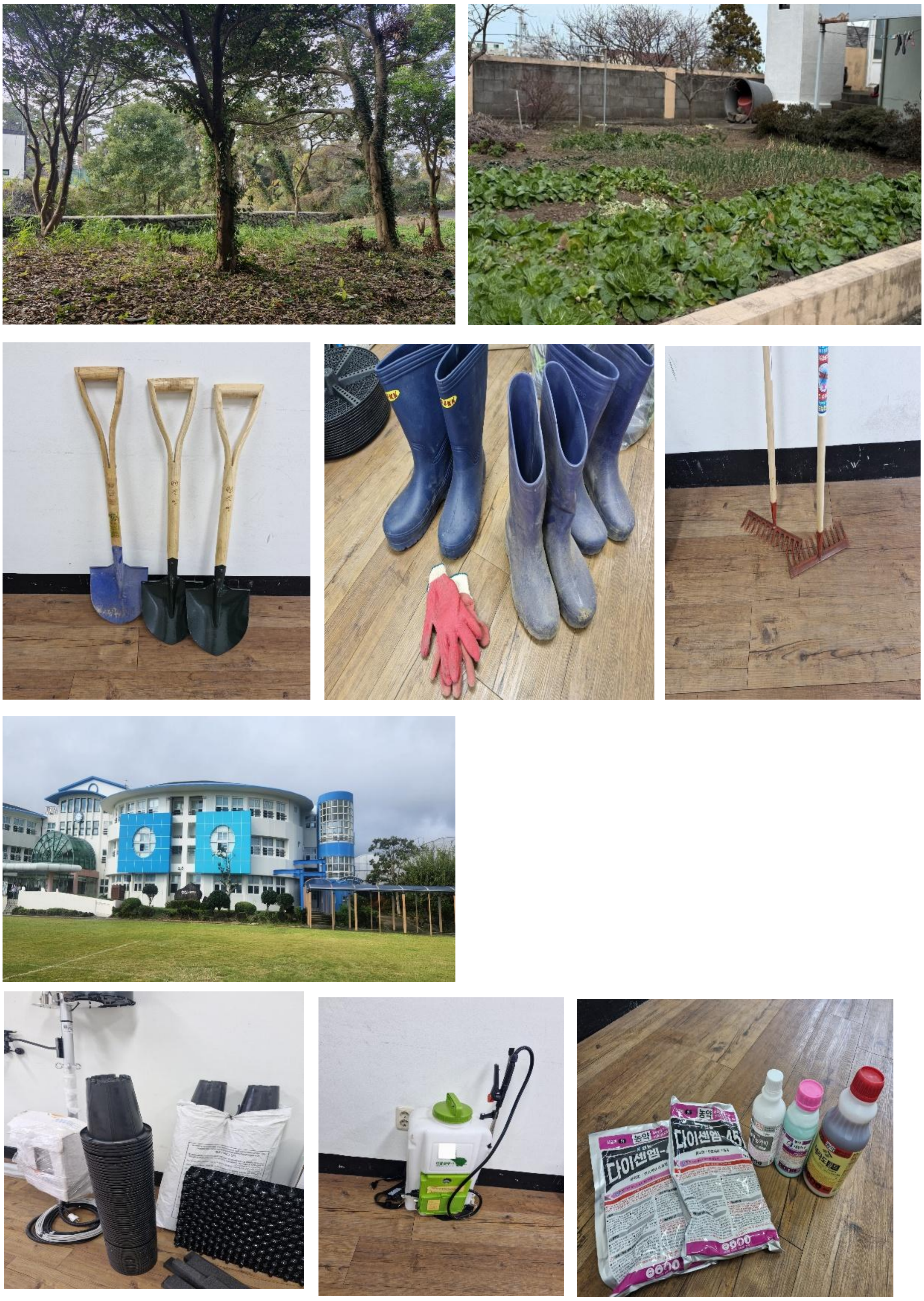

Supplementary Figure 1. Photographs captured by participants to represent experiences of musculoskeletal pain in daily life
